# CIT8, a machine learning-optimized antimicrobial peptide, exhibits potent activity against carbapenem-resistant *Acinetobacter baumannii*

**DOI:** 10.3389/fmicb.2026.1967486

**Published:** 2026-10-19

**Authors:** Charilaos Dellis, Biswajit Mishra, Liyang Zhang, Anindya Basu, Lewis Oscar Felix, Fadi Shehadeh, Narchonai Ganesan, Antonios Kolocouris, Eleftherios Mylonakis

**Affiliations:** 1 Department of Medicine, Houston Methodist Hospital, Houston, TX, United States; 2 Laboratory of Medicinal Chemistry, Section of Pharmaceutical Chemistry, Department of Pharmacy, National and Kapodistrian University of Athens, Athens, Greece; 3 Department of Medicine, Weill Cornell Medical College, New York, NY, United States; 4 School of Pharmaceutical Sciences, Rajiv Gandhi Technological University, Bhopal, India; 5 School of Biomolecular Engineering and Biotechnology, Rajiv Gandhi Technological University, Bhopal, India; 6 Department of Electrical and Computer Engineering, National Technical University of Athens, Athens, Greece

**Keywords:** *Acinetobacter baumannii*, antimicrobial peptides, antimicrobial resistance, carbapenem-resistant *Acinetobacter baumannii*, machine learning, membrane disruption, ML-optimization

## Abstract

As the burden of antimicrobial resistance continues to rise worldwide, the need for novel antimicrobial therapeutics has become increasingly urgent. Machine learning (ML) has emerged as a promising strategy for accelerating antimicrobial peptide discovery; however, experimental characterization remains essential to define the biological properties and broader applicability of ML-optimized peptides. CIT8 is a 13-residue, citropin-based peptide previously developed through ML-optimization with potent antimicrobial activity against methicillin-resistant *Staphylococcus aureus* (MRSA). Here, we demonstrate that its activity extends to multidrug-resistant Gram-negative pathogens. Among these, CIT8 displayed the most potent activity against *Acinetobacter baumannii*, maintaining an MIC of 4 μg/mL against 15 of 16 *A. baumannii* strains tested, including multiple carbapenem-resistant (CRAB) clinical isolates. At 20 μg/mL (5 × MIC), CIT8 reduced an initial population of approximately 5 × 10^7^ CFU/mL of exponential-phase *A. baumannii* to below the limit of detection within 15 min. Consistent with its rapid bactericidal activity, CIT8 induced rapid, dose-dependent membrane depolarization and permeabilization, accompanied by leakage of intracellular ATP. In addition, CIT8 and, even more notably, its D-enantiomer CIT8-D exhibited strong antibiofilm activity while maintaining relatively low mammalian cytotoxicity, with therapeutic indices of >32 for CIT8 in both cell lines and 16.2 and 18.5 for CIT8-D in HaCaT and HKC-8 cells, respectively. Moreover, CIT8 potentiated the bactericidal activity of meropenem against CRAB, with the combination exhibiting bactericidal synergy by time-kill assay. These findings highlight CIT8 as a promising scaffold for further optimization and preclinical evaluation against multidrug-resistant *A. baumannii*.

## 1 Introduction

The rapid rise of antimicrobial resistance (AMR) presents a formidable threat to public health and imposes a substantial economic burden on healthcare systems worldwide. According to the Global Burden of Disease (GBD) study, in 2021, 4.71 million deaths globally were associated with drug-resistant bacterial infections, including 1.14 million deaths directly attributed to AMR (Naghavi et al., 2024). This burden is expected to increase substantially over the coming decades, with annual AMR-associated deaths projected to exceed 8 million and AMR-attributable deaths approaching 2 million by 2050 (O’Neill, 2016; Naghavi et al., 2024). The Gram-negative bacterium *Acinetobacter baumannii* is among the bacterial pathogens driving this global burden because it can rapidly acquire resistance to multiple clinically important antibiotics and can form biofilms, which are difficult to treat (Antunes et al., 2014; Gedefie et al., 2021; Kyriakidis et al., 2021; Murray et al., 2022). Alarmingly, the widespread emergence of carbapenem-resistant *A. baumannii* (CRAB), driven in part by the extensive clinical use of carbapenems for the treatment of multidrug-resistant infections, has dramatically narrowed the available therapeutic options (Nordmann and Poirel, 2019). Recognizing the urgent need for new treatment strategies, the World Health Organization (WHO) has classified CRAB as a critical priority pathogen (World Health Organization, 2017, 2024), while the U.S. Centers for Disease Control and Prevention (CDC) classifies it as an urgent threat (Centers for Disease Control and Prevention, 2019).

Among the alternative therapeutic strategies being explored, antimicrobial peptides (AMPs) have gained considerable attention because of their diverse mechanisms of action and their potential to overcome some of the limitations associated with conventional antibiotics (Zasloff, 2002; Zhang and Gallo, 2016; Ganesan et al., 2023). AMPs are a diverse class of naturally occurring molecules that constitute an essential component of the innate immune system across all domains of life (Ganesan et al., 2023; Santos-Júnior et al., 2024). While AMPs vary greatly in structure and function, they are typically short (10 to 50 amino acids), cationic peptides that often adopt an amphipathic α-helical conformation (Mishra et al., 2022). These characteristics favor interactions with bacterial membranes, often leading to membrane disruption and rapid killing (Ciumac et al., 2019).

In recent years, there has been a growing interest in the use of computational methods, such as machine learning (ML), for the discovery and optimization of AMPs (Das et al., 2021; Santos-Júnior et al., 2024; Wan et al., 2024; Shehadeh et al., 2026; Torres et al., 2026). At the same time, the continued expansion of publicly available AMP databases containing experimentally validated peptide sequences has provided the large datasets needed to develop improved predictive models (Pirtskhalava et al., 2021; Ma et al., 2025; Yao et al., 2025; Wang et al., 2026). Compared with conventional optimization strategies that rely on rational peptide design and experimental screening, computational approaches can efficiently explore much larger regions of peptide sequence space, identify sequence-activity relationships, and prioritize promising candidates for synthesis and experimental evaluation (Huang et al., 2023; Wang et al., 2025). Despite these advances, experimental characterization remains essential to validate the biological properties and broader applicability of ML-optimized peptides (Wan et al., 2024; Sun et al., 2026).

In our previous study (Mishra et al., 2025), we developed an ML-guided peptide optimization strategy that integrates data-driven sequence analysis with traditional structure-based design. As proof of concept, we applied this methodology to a truncated derivative of the naturally occurring α-helical AMP citropin 1.1, which exhibits modest activity against both Gram-positive and Gram-negative bacteria (Wegener et al., 1999; Rodrigues de Almeida et al., 2019), with the aim of improving its efficacy against methicillin-resistant *Staphylococcus aureus* (MRSA). This approach led to the development of CIT8, which displayed substantially enhanced anti-MRSA activity while retaining low cytotoxicity. However, the broader antibacterial spectrum of CIT8 remained largely unexplored. In this study, we investigated the antibacterial activity of CIT8 against multidrug-resistant Gram-negative pathogens, identifying particularly potent activity against *A. baumannii*. In addition, we synthesized its all-D enantiomer, CIT8-D, and examined the underlying mechanism of action, antibiofilm activity, and cytotoxicity of both peptides.

## 2 Materials and methods

### 2.1 Chemicals and reagents

CIT1 (GLFDVIKKVASVI-NH₂), CIT3 (GLFKVIKKVASVI-NH₂), CIT4 (GLFKVIKKVAKVI-NH₂), CIT5 (GLFDVIKKLLSVI-NH₂), CIT7 (GLFKVIKKLLSVI-NH₂), CIT8 (GLFKVIKKLLKVI-NH₂), and CIT8-D (GLFKVIKKLLKVI-NH₂; all chiral residues in the D-configuration) were synthesized by GenScript Inc. (Piscataway, NJ, USA) using solid-phase peptide synthesis and supplied at a reported purity of ≥95%. Peptide purity and molecular weight were confirmed by high-performance liquid chromatography and mass spectrometry, respectively. Peptides were dissolved in distilled deionized water (ddH₂O) to a final concentration of 10 mg/mL, aliquoted, and stored at −80 °C until use. Antibiotics were purchased from Sigma-Aldrich (St. Louis, MO, USA), prepared as 10 mg/mL stock solutions in ddH₂O or dimethyl sulfoxide (DMSO), and stored at −80 °C. Unless otherwise specified, all other chemicals and reagents were purchased from Fisher Scientific (Waltham, MA, USA).

### 2.2 Growth conditions and susceptibility testing

The bacterial strains used in this study (Tables 1, 2) included *A. baumannii* ATCC19606 and ATCC17978 (ATCC, Manassas, VA, USA), 14 clinical *A. baumannii* isolates obtained from the FDA–CDC Antimicrobial Resistance Isolate Bank (Lutgring et al., 2018), *Escherichia coli* K-12 (ATCC, Manassas, VA, USA), *Klebsiella pneumoniae* WGLW2 (BEI Resources, Manassas, VA, USA), *Pseudomonas aeruginosa* PA14 (Rahme et al., 1995), and *Klebsiella aerogenes* EAE 2625. Unless otherwise stated, bacterial strains were routinely cultured in Luria-Bertani broth (LB) (Becton Dickinson, Franklin Lakes, NJ, USA) at 37 °C with shaking at 220 rpm.

**Table 1:** Antibacterial activity of CIT8 (μg/mL) against clinically relevant Gram-negative pathogens.

Compounds	*E. coli* (K-12)	*A. baumannii* (ATCC19606)	*K. pneumoniae* (WGLW2)	*P. aeruginosa* (PA14)	*K. aerogenes* (EAE 2625)
CIT1	>32	>32	>32	32	>32
CIT8	8	4	16	8	16
CIT8-D	4	4	16	8	16
Meropenem	0.5	0.5	0.5	0.5	16
Polymyxin B	1	2	2	1	2
Amikacin	1	2	2	1	4

**Table 2:** Minimum inhibitory concentrations (μg/mL) of CIT8 and CIT8-D against a panel of *A. baumannii* strains.

*A. baumannii strain*	CIT8	CIT8-D	Meropenem	Polymyxin B	Amikacin
ATCC19606	4	4	0.5	2	2
ATCC17978	4	4	0.5	2	1
AR33	4	4	>16	2	8
AR35	4	8	>16	2	4
AR36	4	4	>16	1	8
AR37	4	4	>16	1	8
AR45	4	4	16	2	1
AR52	4	4	8	1	1
AR56	4	8	>16	2	2
AR63	4	4	>16	2	2
AR70	4	4	4	1	1
AR78	4	4	>16	1	>16
AR83	4	4	>16	2	>16
AR88	8	4	>16	2	>16
AR101	4	8	>16	2	>16
AR102	4	4	2	2	>16

The antibacterial activity of all test compounds was determined using the broth microdilution assay according to the Clinical and Laboratory Standards Institute (CLSI) guidelines (Zimmer et al., 2024). In brief, two-fold serial dilutions of each compound were prepared at 10 × the desired final concentration in 10 μL of ddH₂O in sterile 96-well microtiter plates (Corning, Corning, NY, USA). Next, overnight bacterial cultures were diluted in cation-adjusted Mueller-Hinton broth 2 (caMHB; Difco; BD, Franklin Lakes, NJ, USA) and 90 μL of the bacterial suspension was added to each well containing 10 μL of the appropriate compound dilution, resulting in a final bacterial inoculum of approximately 5 × 10^5^ CFU/mL (optical density at 600 nm [OD₆₀₀] ≈ 0.002) and a total volume of 100 μL per well. Following 18 h of incubation at 37 °C, bacterial growth was quantified by measuring the OD₆₀₀ using a SpectraMax 250 microplate reader (Molecular Devices, CA, USA). MICs were defined as the lowest test compound concentration required to prevent detectable bacterial growth (OD₆₀₀ < 0.05). Carbapenem resistance was determined based on meropenem MICs using CLSI susceptibility breakpoints for *A. baumannii* (susceptible, ≤2 μg/mL; intermediate, 4 μg/mL; resistant, ≥8 μg/mL) (Mathers et al., 2026).

To evaluate peptide activity under physiologically relevant conditions, the broth microdilution assays were repeated in the presence of 150 mM NaCl, 2.5 mM CaCl₂, 1 mM MgSO₄, 8 μM ZnCl₂, 5% human serum, or 10% human serum. All susceptibility assays were performed in triplicate with at least three independent experiments, and the most frequently observed MIC was reported.

### 2.3 Killing kinetics of CIT8 against *A. baumannii*

To evaluate bacterial killing, time-kill assays were performed against *A. baumannii* strains ATCC19606 and the carbapenem-resistant clinical isolate AR33. Overnight cultures were diluted 1:10,000 into 25 mL LB in a 50 mL conical tube and incubated at 37 °C with shaking at 225 rpm for approximately 4 h until they reached exponential growth phase. The cells were then collected by centrifugation at 3,500 × g for 5 min, washed three times with equal volumes of phosphate-buffered saline (PBS; Fisher Scientific, MA, USA), and resuspended in PBS to an OD₆₀₀ of approximately 0.4 (≈1 × 10^8^ CFU/mL). Subsequently, 1 mL of the bacterial suspension was mixed with 1 mL of PBS containing the test compounds at twice the desired final concentration in a 96-well assay block (Corning, NY, USA). The resulting final bacterial density corresponded to an OD₆₀₀ of approximately 0.2 (≈5 × 10^7^ CFU/mL). The assay block was sealed with a gas-permeable Breathe-Easy membrane (Millipore Sigma, MO, USA) and incubated at 37 °C with shaking at 225 rpm. At specific time points, 200 μL aliquots were collected, serially diluted tenfold in PBS, and spot-plated onto Mueller-Hinton agar (MHA) plates. After incubating the plates for 18 h at 37 °C, the resulting colonies were counted. The limit of detection was defined as 2 × 10^2^ CFU/mL, corresponding to a minimum of two colonies from a 10 μL spot of the undiluted sample. CIT8 and the control compounds were initially tested at a final concentration of 20 μg/mL, while additional assays were performed with CIT8 at final concentrations of 1, 2, 4, and 8 μg/mL (1/4 ×, 1/2 ×, 1 ×, and 2 × the MIC in caMHB, respectively).

### 2.4 DiSC₃(5) membrane potential assay

Changes in the membrane potential of *A. baumannii* were assessed using the fluorescent dye DiSC₃(5) (3,3′-dipropylthiadicarbocyanine iodide) following a previously established protocol with minor modifications (Mishra et al., 2022). In short, *A. baumannii* ATCC19606 cells were grown to mid-exponential phase, collected by centrifugation, washed three times with PBS, and adjusted to an OD₆₀₀ of 0.1 (≈2.5 × 10^7^ CFU/mL). To energize the bacteria, the suspension was supplemented with 25 mM glucose and incubated at 37 °C with shaking for 15 min. DiSC₃(5) was then added to a final concentration of 1 μM and the mixture was kept in the dark for 10 min. Next, 90 μL of the bacterial suspension was transferred into black, clear-bottom 96-well plates (Corning, NY, USA) and baseline fluorescence was monitored for 30 min using a Cytation 5 multimode plate reader (BioTek, VT, USA) with excitation and emission wavelengths of 620 and 675 nm, respectively. Subsequently, 10 μL of serially diluted peptide or control solution was added to each well, and the fluorescence was recorded every 3 min for 1 h at room temperature. The final test compound concentrations ranged from 1 to 16 μg/mL for CIT8 and polymyxin B and 32 μg/mL for meropenem.

### 2.5 Sytox Green membrane permeability assay

Membrane permeabilization in *A. baumannii* was evaluated by measuring Sytox Green uptake (Kim et al., 2018). Exponential-phase *A. baumannii* ATCC19606 cells were collected, washed three times with PBS, and adjusted to an OD₆₀₀ of 0.2 (≈5 × 10^7^ CFU/mL). Sytox Green (Invitrogen, CA, USA) was then added to a final concentration of 5 μM and the suspension was incubated in the dark for 30 min at room temperature. After incubation, 90 μL of the bacteria-dye suspension was combined with 10 μL of serially diluted peptide or control solution in black, clear-bottom 96-well plates, with final concentrations of 1–16 μg/mL for CIT8 and polymyxin B and 32 μg/mL for meropenem. Fluorescence was measured every 5 min for 2 h at room temperature using a Cytation 3 multimode plate reader (BioTek, VT, USA) at excitation/emission wavelengths of 485/528 nm.

### 2.6 ATP leakage assay

Intracellular ATP leakage from exponential-phase *A. baumannii* cells was quantified using a luciferase-based assay as previously described (Kim et al., 2022). After washing the cells three times with PBS, the bacterial suspension was adjusted to an OD₆₀₀ of approximately 0.2 (≈5 × 10^7^ CFU/mL). Next, 90 μL of the bacterial suspension was combined with 10 μL of each test compound prepared at 10 × the desired final concentration in sterile 96-well plates, and the plates were incubated at 37 °C for 1 h. CIT8 and polymyxin B were tested at final concentrations of 1–16 μg/mL, while meropenem was tested at 32 μg/mL. Following the 1 h incubation, the bacterial cells were pelleted by centrifuging the plates at 4,000 × g for 10 min, and 50 μL of the resulting supernatant was transferred to black 96-well plates. The supernatant was then mixed with an equal volume of BacTiter-Glo reagent (Promega, WI, USA) and incubated in the dark for 5 min at room temperature. Luminescence was subsequently measured using the Cytation 5 multimode plate reader.

### 2.7 Scanning electron microscopy (SEM)

Samples for scanning electron microscopy (SEM) were prepared following a previously described protocol with minor modifications (Shi et al., 2022). *A. baumannii* ATCC19606 cells were grown to mid-exponential phase from an overnight culture. The cells were then washed three times with PBS and adjusted to an OD₆₀₀ of approximately 0.2 (≈5 × 10^7^ CFU/mL). Next, 2 mL of the bacterial suspension was transferred to a microcentrifuge tube and treated with either 20 μg/mL (5 × MIC) CIT8 or ddH₂O for 45 min at 37 °C with shaking. Following treatment, the samples were centrifuged at 14,000 × g for 5 min, the supernatant was discarded, and the cell pellets were fixed with 50 μL of 2.5% glutaraldehyde. After overnight fixation at 4 °C, 30 μL of each sample was deposited onto a (3-aminopropyl) triethoxysilane (APTES)-functionalized Si (100) wafer and incubated for 1 h at room temperature. The wafers were washed three times with PBS, followed by progressive ethanol dehydration of the samples in increasing concentrations (30, 50, 70, 90, and 100% v/v in water) for approximately 15 min at each concentration, with the 100% ethanol step repeated three times. Subsequently, 30 μL of tert-butyl alcohol (50% v/v in ethanol) was applied, and the samples were allowed to air-dry. Once completely dry, the wafers were mounted on aluminum SEM sample holders and coated with a thin Pt/Pd film (7 nm) using a Magnetron 208HR High-Resolution Sputter Coater (Ted Pella Inc., CA, USA). The samples were imaged under high vacuum on a Nova NanoSEM 230 (FEI, OR, USA) with a 5 kV electron beam at the Houston Methodist Research Institute Scanning Electron Microscopy and Atomic Force Microscopy Core. Cell length was quantified using Fiji (ImageJ version 1.54p; Schindelin et al., 2012). The images were calibrated using their corresponding scale bars, and individual cells were measured along their longitudinal axis. Cells with clearly distinguishable endpoints were included, whereas cells partially outside the field of view or substantially obscured by neighboring cells were excluded. In total, 76 untreated and 56 CIT8-treated cells were measured from seven and eleven SEM images, respectively, across two independent experiments. Cell lengths are presented as mean ± standard deviation (SD).

### 2.8 All-atom molecular dynamics (MD) simulations and free energy calculations

All-atom MD simulations were performed following a previously reported protocol (Dellis et al., 2026) with modifications. The initial structure of CIT8 was obtained from the NMR-derived model previously reported by Mishra et al. (2025). To generate the CIT1 structure, the CIT8 model was imported into Maestro (Schrödinger Release, 2026: Maestro, 2026) and targeted amino acid substitutions corresponding to the CIT1 sequence were performed. Peptide topology files were generated using the CHARMM-GUI Force Field Converter (Lee et al., 2016, 2022) with the CHARMM36m force field (Huang et al., 2017).

To model the Gram-negative bacterial membrane, a lipid bilayer composed of 1-palmitoyl-2-oleoyl-*sn*-glycero-3-phosphoethanolamine (POPE) and 1-palmitoyl-2-oleoyl-*sn*-glycero-3-phospho-(1′-*rac*-glycerol) (POPG) at a 3:1 molar ratio was constructed. The lipid bilayer and the simulation box were generated using the CHARMM-GUI Membrane Builder (Jo et al., 2007, 2009; Wu et al., 2014) and the CHARMM36 force field (Klauda et al., 2010), with NaCl added at a concentration of 0.15 M. Each system contained approximately 92,000 atoms, including 36,800 lipid atoms (294 lipid molecules) and 55,200 water atoms (18,400 water molecules). The final membrane simulation box measured 92 × 92 × 105 Å and all simulations were performed using the GROMACS 2025.4 software package (Abraham et al., 2015). Before the simulations, the membrane-only system was minimized for 5,000 steps using the steepest descent method (Wardi, 1988) and subsequently equilibrated through two consecutive NVT steps, followed by four NPγT steps. In all simulations, a 12 Å cutoff was applied for short-range electrostatic and van der Waals interactions, while all bonds involving hydrogen atoms were constrained using the LINCS algorithm (Hess et al., 1997). The temperature was maintained at 310 K with the modified Berendsen (v-rescale) thermostat (Bussi et al., 2007) and a temperature-coupling constant of 1.0 ps. In the NPγT stages, the pressure was controlled using the stochastic cell-rescaling (C-rescale) barostat (Bernetti and Bussi, 2020) with surface-tension coupling, a target normal pressure of 1 bar, and a pressure-coupling time constant of 5.0 ps. During equilibration, positional restraints of 2.5 kcal mol^−1^ Å^−2^ were applied to the lipid headgroups and the restraint force constant was progressively reduced to allow gradual membrane relaxation while preserving bilayer integrity. After the equilibration phase, an unrestrained NPγT production simulation was performed for 500 ns and its final frame was used as the starting configuration for each peptide-membrane system. The GROMACS insert-molecules utility was then used to place a single CIT1 or CIT8 peptide in the aqueous phase above the membrane headgroups, and each system was equilibrated for 250 ps. Finally, two independent 1 μs production simulations were performed for each peptide-membrane system using randomized initial velocities. Trajectories were visualized using Visual Molecular Dynamics (VMD) (Humphrey et al., 1996) and analyzed using GROMACS tools and custom Python scripts based on the MDAnalysis library (Michaud-Agrawal et al., 2011; Gowers et al., 2016). Peptide secondary structure was assigned using the Define Secondary Structure of Proteins (DSSP) algorithm implemented in MDTraj (McGibbon et al., 2015), and peptide orientation was quantified as the angle between the vector connecting the N- and C-terminal Cα atoms and the membrane normal (z-axis).

The free energy profile of CIT8 was characterized using steered molecular dynamics followed by potential of mean force (PMF) calculations with umbrella sampling (US/PMF). Starting from the equilibrated peptide-membrane system, CIT8 was pulled along the membrane normal using a harmonic restraint of 1,000 kJ mol^−1^ nm^−2^ and a constant pulling velocity of 0.00005 nm ps^−1^. Umbrella sampling windows were then extracted at 0.2 nm intervals along the reaction coordinate, and each window underwent 1 ns of equilibration followed by a 50 ns production simulation. The PMF curve was reconstructed using the Weighted Histogram Analysis Method (WHAM) free energy estimator, implemented in GROMACS (Kumar et al., 1992; Hub et al., 2010). Statistical uncertainty was estimated using 200 trajectory-based bootstrap replicates with autocorrelation correction. Convergence was assessed by recalculating the PMF using progressively increasing portions (10, 20, 30, 40, and 50 ns) of the umbrella sampling trajectories and evaluating the overlap between neighboring umbrella sampling windows from the distributions of the sampled reaction coordinate.

### 2.9 Biofilm formation assay

The inhibitory activity of CIT8 and CIT8-D against *A. baumannii* biofilm formation was evaluated using a previously described protocol with modifications (Kamoshida et al., 2022). Overnight cultures of *A. baumannii* ATCC19606 and AR33 were adjusted in LB to an OD₆₀₀ of 0.01 (≈2.5 × 10^6^ CFU/mL). Then, 90 μL of each bacterial suspension was added to the wells of a 96-well plate containing 10 μL of serially diluted peptide solutions or controls at 10 × the desired concentration, and the plates were incubated at 37 °C under static conditions for 24 h. The final concentrations of all tested compounds ranged from 2 to 64 μg/mL. After incubation, the wells were gently washed with PBS to remove non-adherent cells. To assess biofilm viability, 100 μL of LB containing 20% of the metabolic dye CyQUANT™ XTT (Fisher Scientific, MA, USA) was added to each well (Kaushik et al., 2016), and the plates were incubated for 2 h at 37 °C before absorbance was measured at 450 nm using a SpectraMax 250 plate reader. Biofilm metabolic activity, used as an indirect indicator of cell viability, was expressed as a percentage of the untreated bacterial control based on the XTT absorbance.

### 2.10 Established biofilm assay

To assess the effects of CIT8 and CIT8-D against established *A. baumannii* ATCC19606 and AR33 biofilms, bacterial suspensions were prepared as described above to an OD₆₀₀ of 0.01 (≈2.5 × 10^6^ CFU/mL), and 90 μL of each suspension was added to the wells of a 96-well plate. Following incubation under static conditions at 37 °C for 24 h, the medium was removed, and the wells were gently washed with PBS to remove loosely attached cells. The established biofilms were then treated with 100 μL of PBS containing serially diluted peptides or control solutions for an additional 24 h. The final concentrations of all tested compounds ranged from 2 to 64 μg/mL. After the second incubation, the wells were again washed with PBS to remove non-adherent cells, and the viability of biofilm-associated cells was indirectly assessed using the CyQUANT™ XTT assay as described above.

In addition, the effects of the two peptides on established AR33 biofilm biomass were evaluated following a previously reported protocol (Kong et al., 2022). In brief, established biofilms were prepared and treated as described above with CIT8, CIT8-D, meropenem, or polymyxin B at concentrations ranging from 8 to 64 μg/mL for 24 h. After treatment, the wells were washed with PBS and stained with 0.1% crystal violet for 15 min. Excess stain was then removed by washing with PBS, and the retained crystal violet was solubilized with 95% ethanol. Absorbance was measured at 595 nm using a SpectraMax 250 plate reader, and the remaining biofilm biomass was expressed as a percentage of the untreated control.

### 2.11 Fluorescence microscopy of established biofilms

To visualize the effects of CIT8 and CIT8-D on established *A. baumannii* biofilms, fluorescence microscopy was performed following a previously described protocol (Dellis et al., 2026) with minor modifications. An overnight culture of *A. baumannii* ATCC19606 was diluted in LB medium to an OD₆₀₀ of 0.01 (≈2.5 × 10^6^ CFU/mL), and 500 μL of the bacterial suspension was added to the chambers of Lab-Tek™ Chambered Coverglass (Thermo Fisher Scientific, MA, USA). To allow biofilm formation, the chambers were incubated under static conditions at 37 °C for 24 h. The chambers were then gently washed with PBS, and 500 μL of either PBS (untreated control) or PBS containing CIT8, CIT8-D, or meropenem (each at 40 μg/mL) was added. After 24 h of treatment at 37 °C, the chambers were washed with PBS, and the biofilms were stained using the LIVE/DEAD™ BacLight™ Bacterial Viability Kit (Invitrogen, CA, USA) according to the manufacturer’s instructions. Biofilms were visualized using an Olympus Fluoview™ FV3000 microscope (Olympus, Tokyo, Japan).

### 2.12 *In vitro* cytotoxicity

Cytotoxicity of CIT8 and CIT8-D toward human keratinocyte HaCaT cells and human kidney cortex HKC-8 cells (AddexBio, San Diego, CA, USA) was assessed using the CyQUANT™ XTT Cell Viability Assay Kit. The cells were cultured at 37 °C with 5% CO₂ in Dulbecco’s Modified Eagle Medium (DMEM; Fisher Scientific, MA, USA) supplemented with 10% heat-inactivated fetal bovine serum and 1% penicillin/streptomycin. At approximately 85% confluence, cells were harvested and the suspension was adjusted to a concentration of 1 × 10^6^ cells/mL. Next, 100 μL of the cell suspension was seeded into the wells of a 96-well plate (1 × 10^5^ cells/well), and the plates were incubated for 24 h at 37 °C with 5% CO₂. The medium was carefully aspirated and replaced with 100 μL of fresh DMEM containing two-fold serial dilutions of CIT8 or CIT8-D at final concentrations ranging from 4 to 128 μg/mL. Control wells received 100 μL of fresh DMEM without peptide. Following 24 h of treatment, 70 μL of the XTT-electron coupling reagent mixture was added to each well and the plates were incubated for 4 h, after which absorbance was measured at 450 and 660 nm using a SpectraMax 250 plate reader. Cell viability was calculated according to the manufacturer’s instructions. The median lethal concentration (LC₅₀) was defined as the peptide concentration that reduced mammalian cell viability by 50% and was determined from the concentration-response curve. To provide a concentration-based estimate of *in vitro* selectivity between mammalian cytotoxicity and antibacterial activity, the therapeutic index (TI) was calculated as the ratio of LC₅₀ to the MIC against *A. baumannii* ATCC19606 under standard assay conditions (LC₅₀/MIC), as previously described (Jiang et al., 2014; Rawson et al., 2022).

### 2.13 Checkerboard assay

Checkerboard microdilution assays were used to assess the interaction between CIT8 and conventional antibiotics (Doern, 2014). Two-fold serial dilutions of CIT8 (10 μL) were combined with 10 μL of two-fold serial dilutions of each antibiotic in a 96-well plate to generate an 8 × 8 concentration matrix. Each compound was prepared at 10 × its intended final concentration before addition to the wells. An overnight culture of either *A. baumannii* ATCC19606 or AR33 was then diluted in caMHB, and 80 μL of the bacterial suspension was added to each well, resulting in a final bacterial inoculum of approximately 5 × 10^5^ CFU/mL in a total volume of 100 μL. The plates were incubated for 18 h at 37 °C, and bacterial growth was measured by recording the OD₆₀₀ using a SpectraMax 250 plate reader. The fractional inhibitory concentration index (FICi) was calculated as:

$$\begin{array}{l} \text{FICi}=({\mathrm{MIC}}_{A\;\text{in combination}}/{\mathrm{MIC}}_{A\;\text{alone}})\\ +({\mathrm{MIC}}_{B\;\text{in combination}}/{\mathrm{MIC}}_{B\;\text{alone}}) \end{array}$$

Interactions were interpreted as follows: FICi ≤ 0.5, synergy; 0.5 < FICi ≤ 4, no interaction; and FICi > 4, antagonism (Odds, 2003).

### 2.14 Combination time-kill assay

To assess the bactericidal interaction between CIT8 and meropenem, combination time-kill assays were performed against the CRAB isolates AR33, AR36, and AR63. Exponential-phase bacterial cells were prepared as described in Section 2.3. CIT8 and meropenem working solutions were prepared at 4 × the desired final concentration, and 250 μL of each solution was combined in a 96-well assay block. For individual agent treatments, the corresponding second agent was replaced with an equal volume of PBS. Subsequently, 500 μL of the bacterial suspension was added to each treatment, resulting in a final assay volume of 1 mL containing approximately 5 × 10^7^ CFU/mL and CIT8 at 1, 2, 4, or 8 μg/mL, either alone or in combination with 40 μg/mL meropenem. Meropenem (40 μg/mL) alone and an untreated control were included for comparison. After 4 h of treatment, bacterial viability was determined as described in Section 2.3. Bactericidal synergy was defined as a ≥ 2-log₁₀ CFU/mL reduction in viable bacterial counts for the combination compared with the most active individual agent (Eliopoulos and Moellering, 1996).

### 2.15 Data analysis

Statistical analysis was performed using GraphPad Prism (version 10.12). Unless otherwise specified, n denotes the number of independent experiments; technical replicates were averaged within each independent experiment before statistical analysis. For experiments comparing multiple treatment groups with a single untreated control, one-way ANOVA followed by Dunnett’s multiple comparisons test was used. For the combination experiments, one-way ANOVA followed by Šídák’s multiple comparisons test was used to compare each combination treatment with the corresponding individual-agent treatment, as indicated in the figure. Significance thresholds were defined as follows: *p < 0.05, **p < 0.01, ***p < 0.001, and ****p < 0.0001. For SEM cell-length measurements, the distributions of individual cell lengths were compared using a two-tailed Mann–Whitney U test. This cell-level analysis was considered exploratory, as image acquisition primarily focused on identifying membrane-associated morphological alterations rather than systematic morphometric sampling, and the test did not account for clustering of cells within images or independent experiments. For biofilm and mammalian cell viability assays, concentration-response data were normalized to the corresponding untreated control and analyzed by nonlinear regression using a variable-slope inhibition model. The concentrations producing a 50% reduction in normalized response were estimated from the fitted curves, with 95% confidence intervals determined using the profile-likelihood method. When a 50% response was not reached within the tested concentration range or could not be reliably estimated by nonlinear regression, a 50% effect concentration was not reported.

## 3 Results

### 3.1 Antibacterial activity of CIT8 against Gram-negative bacteria

As CIT8 had previously demonstrated potent anti-staphylococcal activity (MIC of 4 μg/mL against MRSA MW2), we initially examined its antibacterial activity against five major Gram-negative pathogens, including *E. coli* K-12, *A. baumannii* ATCC19606, *K. pneumoniae* WGLW2, *P. aeruginosa* PA14 and *K. aerogenes* EAE 2625 (Table 1). CIT8 displayed antibacterial activity against all five Gram-negative pathogens, with an MIC of 4 μg/mL against *A. baumannii* ATCC19606 and MIC values ranging between 8 and 16 μg/mL against the other pathogens. For comparison, we included CIT1, which served as a reference peptide and displayed little to no activity against all five Gram-negative species at concentrations up to 32 μg/mL, as well as CIT8-D, which displayed an antibacterial profile comparable to that of CIT8. To further evaluate the antibacterial activity of CIT8 and CIT8-D against *A. baumannii*, we tested their activity against a panel of multidrug-resistant clinical isolates including several carbapenem-resistant strains from the FDA–CDC Antimicrobial Resistance Isolate Bank (Table 2). Both peptides retained potent antibacterial activity against all clinical isolates tested. CIT8 exhibited an MIC of 4 μg/mL against 15 of 16 strains and 8 μg/mL against one strain, whereas CIT8-D exhibited MIC values of 4 μg/mL against 13 strains and 8 μg/mL against three strains.

Given the marked difference in activity between CIT1 and CIT8 against *A. baumannii*, we examined the contribution of the four amino acid substitutions distinguishing the two peptides (Supplementary Table S1). Replacement of the negatively charged aspartic acid at position 4 with the positively charged lysine (D4K; CIT3) moderately increased antibacterial activity, reducing the MIC to 16 μg/mL. Further increasing the net positive charge by replacing the polar, uncharged serine at position 11 with lysine (D4K and S11K; CIT4) did not improve activity beyond that of CIT3, with the MIC remaining at 16 μg/mL. The substitutions affecting hydrophobicity alone showed no noticeable improvement, as replacement of valine and alanine at positions 9 and 10, respectively, by leucine (V9L and A10L; CIT5) resulted in an MIC >32 μg/mL. However, when the charge- and hydrophobicity-altering substitutions were introduced together (D4K, V9L and A10L; CIT7), the MIC decreased to 4 μg/mL, matching that of CIT8.

We next examined the effect of physiological salts and human serum on the antibacterial activity of CIT8 and CIT8-D against *A. baumannii* (Table 3). In the presence of 150 mM NaCl, the antibacterial activity of CIT8 was unaffected, with the MIC remaining at 4 μg/mL. By comparison, addition of 8 μM ZnCl₂ resulted in a 2-fold increase in MIC to 8 μg/mL, while 2.5 mM CaCl₂ and 1 mM MgSO₄ resulted in a 4-fold increase to 16 μg/mL. Similarly, the MIC increased by 2-fold in the presence of 5% human serum and 4-fold in the presence of 10% human serum. This trend was observed for both *A. baumannii* ATCC19606 and the CRAB isolate AR33. CIT8-D displayed a similar activity profile, with the only notable differences occurring in the presence of NaCl for ATCC19606 and MgSO₄ and 10% human serum for AR33.

**Table 3:** Minimum inhibitory concentrations (μg/mL) of CIT8 and CIT8-D against *A. baumannii* in the presence of physiological salt concentration or human serum.

*A. baumannii* strain	Compound	MIC (μg/mL) in the absence of salts	MIC (μg/mL) in the presence of salts or serum					
			NaCl (150 mM)	CaCl₂ (2.5 mM)	MgSO₄ (1 mM)	ZnCl₂ (8 μM)	Serum (5%)	Serum (10%)
ATCC19606	CIT8	4	4	16	16	8	8	16
	CIT8-D	4	8	16	16	8	8	16
	Polymyxin B	2	2	2	4	2	4	2
AR33	CIT8	4	4	16	16	8	8	16
	CIT8-D	4	4	16	8	8	8	8
	Polymyxin B	2	2	2	2	2	2	2

### 3.2 CIT8 rapidly kills *A. baumannii*

Since most antimicrobial peptides exhibit bactericidal properties, we sought to determine whether CIT8 displayed similar killing kinetics against *A. baumannii* using time-kill assays (Figure 1). Treatment with 20 μg/mL (5 × MIC) CIT8 reduced viable bacterial counts from an initial inoculum of approximately 5 × 10^7^ CFU/mL to below the limit of detection within 15 min for both *A. baumannii* ATCC19606 (Figure 1A) and AR33 (Figure 1B). Although polymyxin B had a lower MIC (2 μg/mL), it reduced bacterial counts below the limit of detection after 30 min for ATCC19606 and 120 min for AR33. In contrast, treatment with 20 μg/mL meropenem did not reduce bacterial counts in either strain during the 4 h experimental period.

**Figure 1:**
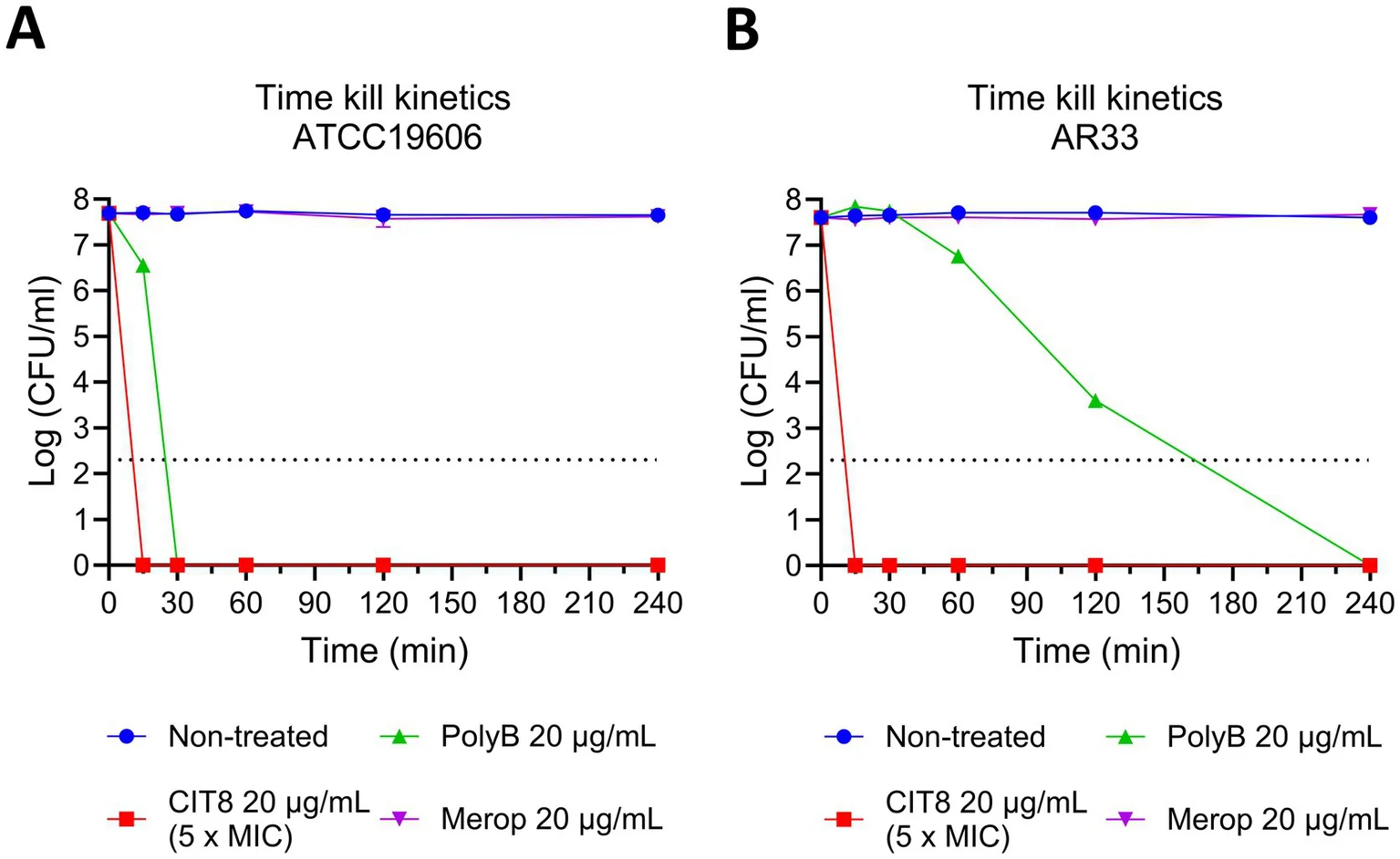
CIT8 demonstrates bactericidal activity against *Acinetobacter baumannii*. **(A,B)** Killing kinetics of CIT8 against exponential-phase *A. baumannii* ATCC19606 **(A)** and AR33 cells **(B)** at 20 μg/mL (5 × MIC). Polymyxin B (PolyB, 20 μg/mL) and meropenem (Merop, 20 μg/mL) were included as antibiotic controls, while an untreated group served as a bacterial control. The dashed line indicates the limit of detection (2 × 10^2^ CFU/mL). Data are presented as mean ± SD of three independent experiments each performed in three technical replicates (*n* = 3).

Additional time-kill assays showed that CIT8-mediated killing was concentration-dependent (Supplementary Figure S1). Treatment with 1 and 2 μg/mL (1/4 × and 1/2 × MIC, respectively) had limited effects on bacterial viability within 4 h, whereas 4 μg/mL (1 × MIC) reduced viable bacterial counts by approximately 0.8 and 1.5 log₁₀ CFU/mL in ATCC19606 and AR33, respectively. At 8 μg/mL (2 × MIC), CIT8 exhibited substantially greater killing, reducing ATCC19606 bacterial counts by approximately 5 log₁₀ CFU/mL within 60 min and decreasing AR33 bacterial counts below the limit of detection within 2 h.

### 3.3 CIT8 compromises membrane integrity in *A. baumannii*

To investigate the mechanism underlying the rapid bactericidal activity of CIT8, we first examined its effect on the membrane potential of *A. baumannii* using the DiSC₃(5) dye. Upon addition of CIT8, we observed a rapid dose-dependent increase in fluorescence, indicating membrane depolarization (Figure 2A). Notably, fluorescence increased even at sub-MIC concentrations of 1 μg/mL (1/4 × MIC) and 2 μg/mL (1/2 × MIC). Similarly, polymyxin B triggered a rapid dose-dependent increase in fluorescence at concentrations as low as 4 μg/mL (2 × MIC), whereas meropenem had no detectable effect at concentrations up to 32 μg/mL (64 × MIC).

**Figure 2:**
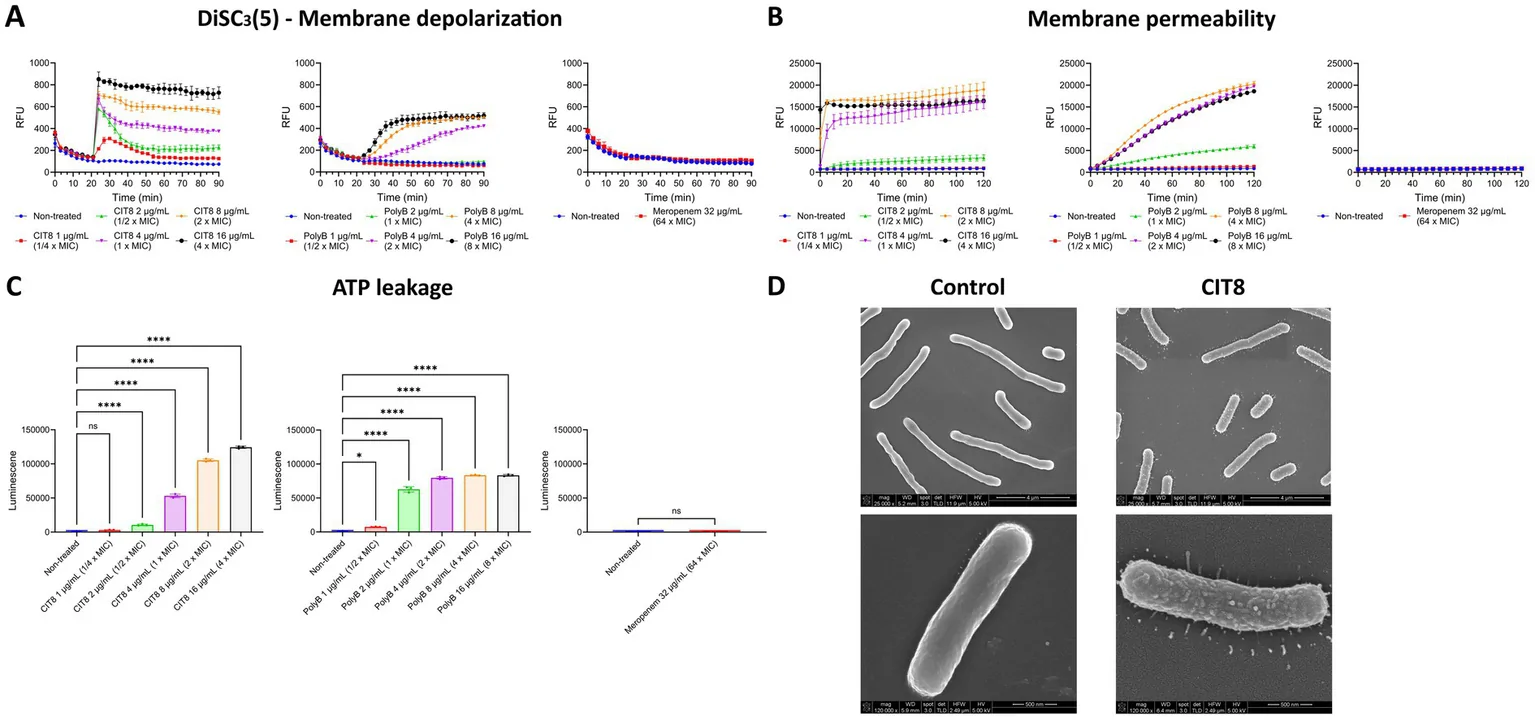
CIT8 disrupts the membrane integrity of *A. baumannii*. **(A)** Membrane depolarization of exponential-phase *A. baumannii* ATCC19606 cells following treatment with CIT8, polymyxin B (PolyB) or meropenem at the indicated concentrations, measured using the membrane potential-sensitive dye DiSC₃(5). Fluorescence was monitored for 30 min before compound addition to establish a stable baseline, after which compounds were added and fluorescence was continuously recorded for 1 h. **(B)** Membrane permeabilization of exponential-phase *A. baumannii* ATCC19606 cells following treatment with CIT8, polymyxin B (PolyB) or meropenem at the indicated concentrations, measured using the membrane-impermeable DNA-binding dye Sytox Green. Fluorescence was monitored for 2 h. **(C)** Leakage of intracellular ATP from exponential-phase *A. baumannii* ATCC19606 cells following treatment with CIT8, polymyxin B (PolyB) or meropenem at the indicated concentrations for 1 h. Data in **(A,B)** are presented as mean ± SD of three technical replicates from a representative experiment. Experiments were independently repeated three times using three technical replicates per experiment. Data in **(C)** are presented as mean ± SD of three independent experiments each performed in three technical replicates (*n* = 3). Statistical significance in **(C)** was determined by one-way ANOVA followed by Dunnett’s multiple comparisons test, with the untreated group as the control (ns, not significant; *p < 0.05; ****p < 0.0001). **(D)** Representative scanning electron micrographs of exponential-phase *A. baumannii* ATCC19606 cells following treatment with 20 μg/mL CIT8 (right panels) or ddH₂O (left panels) for 45 min. Images were acquired at 25,000 × (upper panels) and 120,000 × (lower panels) magnification. The experiment was independently repeated twice, with two technical replicates per experiment.

We next examined whether membrane depolarization induced by CIT8 was accompanied by membrane permeabilization using the membrane-impermeable DNA-binding fluorescent dye Sytox Green. After exposing *A. baumannii* cells to CIT8, fluorescence increased immediately, followed by a plateau (Figure 2B). Similar to the DiSC₃(5) assay, the response was dose-dependent and remained evident even at a sub-MIC concentration of 2 μg/mL (1/2 × MIC). In comparison, polymyxin B induced a more gradual increase in fluorescence that did not reach a plateau during the 2 h experiment. Meropenem, which does not target the bacterial membrane, did not increase fluorescence above baseline.

Having established that CIT8 compromises the membrane integrity of *A. baumannii*, we employed a luciferase-based bioluminescence assay to assess whether it also causes leakage of intracellular ATP. Treatment with CIT8 for 1 h resulted in a significant increase in luminescence (*p* < 0.0001) at concentrations as low as 2 μg/mL (1/2 × MIC) (Figure 2C). Likewise, polymyxin B induced increased luminescence at concentrations ≥ 1 μg/mL (1/2 × MIC), while meropenem had no significant effect.

To visualize morphological changes associated with membrane damage, we examined *A. baumannii* ATCC19606 cells using scanning electron microscopy. Compared with the untreated control, cells exposed to 20 μg/mL (5 × MIC) CIT8 for 45 min displayed a heterogeneous, roughened cell surface with surface indentations and extracellular debris (Figure 2D). Quantitative analysis yielded mean cell lengths of 3.14 ± 1.69 μm for untreated cells and 2.83 ± 1.49 μm for CIT8-treated cells, with no statistically significant difference between the measured cell-length distributions (two-tailed Mann–Whitney U test, *p* = 0.36). Collectively, these findings suggest that the antibacterial activity of CIT8 against *A. baumannii* involves a membrane-targeting mechanism of action.

### 3.4 Membrane-binding behavior of CIT8 revealed by molecular dynamics simulations

To delve into the membrane-binding behavior of CIT8 at the molecular level, we performed two independent 1 μs all-atom MD simulations using a POPE: POPG (3:1) lipid bilayer model and included CIT1 for comparison (Figure 3A). In both systems, the peptides rapidly bound to the membrane surface and remained associated with the lipid bilayer throughout the simulations (Figure 3B). Although CIT8 remained slightly closer to the bilayer center (19.9 ± 5.7 Å and 20.8 ± 6.3 Å) than CIT1 (22.0 ± 6.0 Å and 21.1 ± 2.7 Å) across both simulations, neither peptide penetrated the hydrophobic membrane core but instead localized at the membrane interface (Figure 3C, Supplementary Table S2). Residue-specific analysis revealed that residues 9–13 of CIT8 generally remained closer to the bilayer center and exhibited higher membrane contact occupancy than the corresponding residues of CIT1 (Supplementary Figure S2). Furthermore, CIT8 exhibited higher POPG contact occupancy across much of its sequence (Supplementary Table S3). The most pronounced difference was observed at position 4, where the mean POPG contact occupancy increased from 36.0 ± 3.5% for Asp4 in CIT1 to 81.0 ± 7.7% for Lys4 in CIT8.

**Figure 3:**
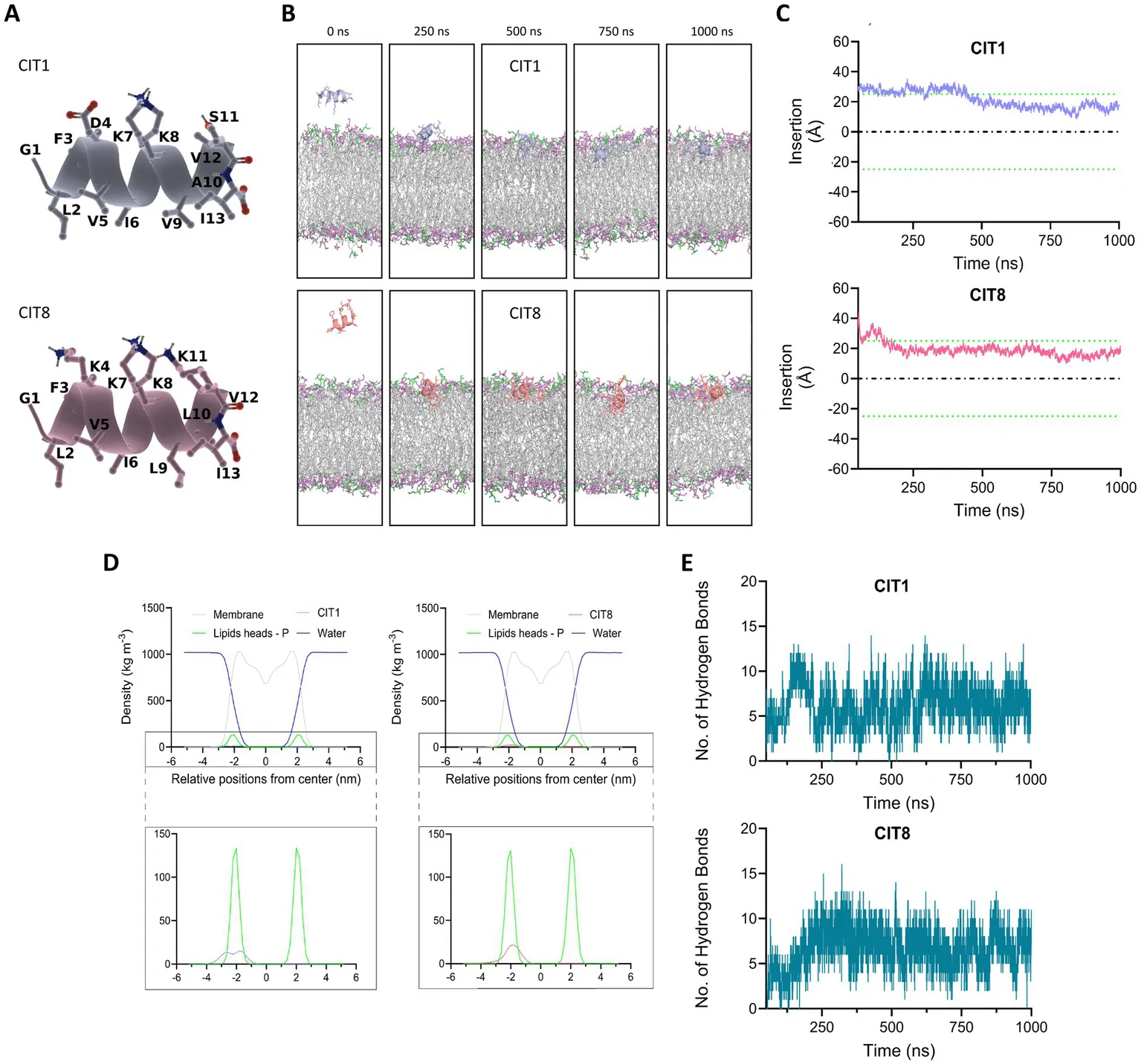
Characterization of the membrane-binding behavior of CIT8 using molecular dynamics simulations. **(A)** Initial structures of CIT1 and CIT8 used for molecular dynamics simulations. **(B)** Representative snapshots showing the interaction of CIT1 (colored light blue, upper panels) and CIT8 (colored pink, lower panels) with the POPE: POPG (3:1) lipid bilayer during the 1 μs simulations. POPE lipid headgroups are shown in purple and POPG lipid headgroups in green. **(C)** Representative peptide insertion profiles relative to the membrane center over the course of the simulations. The dashed black line indicates the membrane center, while the green dashed lines indicate the average positions of the lipid phosphate groups. **(D)** Representative mass density profiles showing the distributions of the lipids (Membrane), lipid phosphate groups (Lipid heads P), water molecules and peptides relative to the membrane center. **(E)** Number of peptide–lipid hydrogen bonds formed throughout the simulations. Data in **(B–E)** are representative of two independent 1 μs simulations.

Density analysis of the all-atom MD simulations further demonstrated that both peptides predominantly localized within the lipid headgroup region, with their density distributions closely aligning with the phosphate density profile (Figure 3D). Consistent with these observations, both peptides maintained persistent hydrogen bonds with the lipid headgroups throughout the simulations, averaging approximately 5–6 hydrogen bonds across all runs (Figure 3E, Supplementary Table S2). To further characterize the membrane interaction of CIT8, we calculated the potential of mean force (PMF) associated with its movement from the aqueous phase toward the center of the POPE: POPG bilayer. The resulting PMF revealed a free energy minimum of approximately −2.69 kcal mol^−1^ at a reaction coordinate of ~28.3 Å from the bilayer center, corresponding to the membrane interface (Supplementary Figure S3A). From this minimum, the free energy increased progressively as the peptide was displaced toward the bilayer center, reaching approximately 28 kcal mol^−1^ within the hydrophobic core. The PMF profiles became increasingly consistent with longer sampling times, while the distributions of neighboring umbrella sampling windows showed adequate overlap along the reaction coordinate (Supplementary Figures S3B,C). Together, these results suggest that migration from the membrane interface into the bilayer interior is energetically unfavorable.

Despite their similar membrane interaction profiles, CIT8 and CIT1 differed in their conformational behavior. Secondary-structure analysis using the DSSP algorithm showed that CIT8 retained a continuous α-helical core from Lys4 to Leu10 throughout the simulations, while the N-terminus (Gly1) and C-terminus (Lys11-Ile13) remained more flexible (Supplementary Figure S4). In contrast, CIT1 exhibited a less persistent α-helical structure, with residues toward the C-terminus, particularly Val9-Ile13, more frequently adopting irregular conformations. These differences were reflected in the average α-helical content, which ranged from 31.7 to 64.4% for CIT8 compared with 17.9 to 38.2% for CIT1 (Supplementary Table S2).

### 3.5 Activity of CIT8 and CIT8-D against *A. baumannii* biofilm

We next evaluated the effects of CIT8 and CIT8-D on biofilms formed by *A. baumannii* ATCC19606 and AR33. When added during biofilm formation, neither peptide substantially affected the XTT signal at concentrations of 2 μg/mL or lower. CIT8 decreased cell viability by 85% at 8 μg/mL and by >95% at concentrations of 16 μg/mL or higher in both strains. CIT8-D exhibited slightly greater activity, achieving a > 95% reduction in cell viability at 4 μg/mL and 8 μg/mL in ATCC19606 and AR33, respectively (Figure 4A).

**Figure 4:**
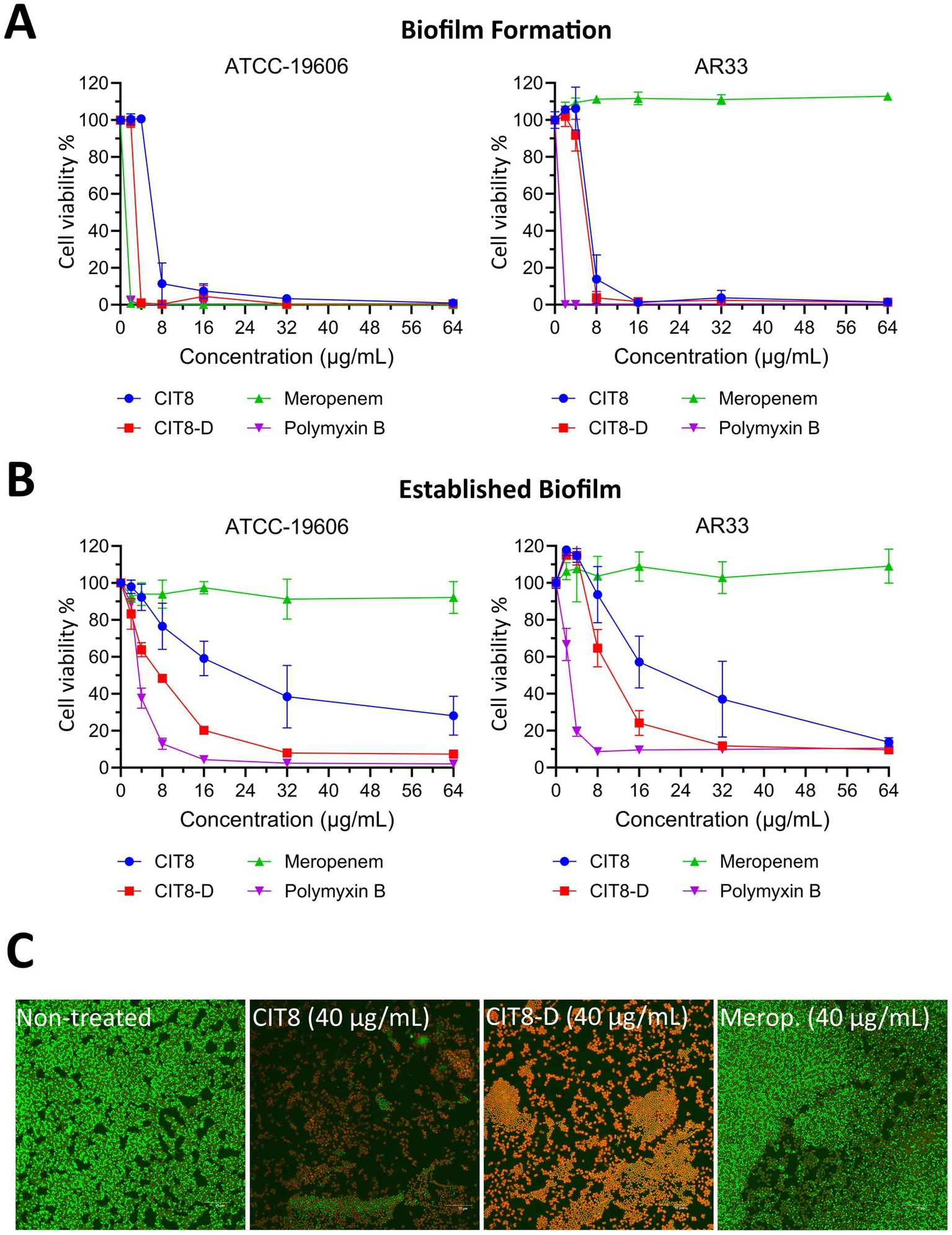
Activity of CIT8 and CIT8-D against *A. baumannii* biofilms. **(A)** Effect of CIT8, CIT8-D, polymyxin B, or meropenem on *A. baumannii* ATCC19606 (left panel) or AR33 (right panel) during biofilm formation. Cell viability was indirectly assessed using the XTT metabolic activity assay after 24 h of treatment at the indicated concentrations. **(B)** Effect of CIT8, CIT8-D, polymyxin B, or meropenem against *A. baumannii* ATCC19606 (left panel) or AR33 (right panel) biofilms established for 24 h. Cell viability was indirectly assessed using the XTT metabolic activity assay after 24 h of treatment at the indicated concentrations. Data in **(A,B)** are presented as mean ± SD of three independent experiments each performed in three technical replicates (*n* = 3). **(C)** Representative fluorescence microscopy images of 24 h established *A. baumannii* ATCC19606 biofilms following treatment with PBS, 40 μg/mL CIT8, 40 μg/mL CIT8-D or 40 μg/mL meropenem (from left to right) for 24 h. Biofilms were stained with SYTO9 (green, live cells) and propidium iodide (red, dead cells) for 15 min before imaging. The experiment was independently repeated twice, with two technical replicates per experiment.

Against established biofilms, both peptides exhibited concentration-dependent antibiofilm activity. After 24 h of treatment, CIT8 reduced biofilm-associated cell viability by 50% at 23.5 μg/mL (95% CI, 18.9–29.7 μg/mL) for ATCC19606 and 22.6 μg/mL (95% CI, 17.4–29.7 μg/mL) for AR33. Notably, CIT8-D exhibited greater antibiofilm activity, achieving the same reduction at 6.6 μg/mL (95% CI, 6.0–7.2 μg/mL) and 10.8 μg/mL (95% CI, 9.0–13.1 μg/mL), respectively, and reducing biofilm-associated cell viability by ≥90% at ≥32 μg/mL in both strains (Figure 4B). Both peptides also reduced established AR33 biofilm biomass, although to a lesser extent, with reductions of approximately 46 and 56% for CIT8 and CIT8-D, respectively, at 64 μg/mL (Supplementary Figure S5). Among the antibiotic controls, meropenem showed no activity against established biofilms, whereas polymyxin B exhibited potent activity against biofilm-associated cells across the concentration range tested and reduced AR33 biofilm biomass to a similar extent as CIT8 and CIT8-D (≈47% at 64 μg/mL; Figure 4B and Supplementary Figure S5).

To validate these findings, established *A. baumannii* ATCC19606 biofilms were treated with CIT8, CIT8-D, or meropenem at 40 μg/mL and stained with a combination of SYTO9 (live-cell indicator) and propidium iodide (PI; dead-cell indicator) for fluorescence microscopy (Figure 4C). Untreated biofilms and those exposed to meropenem showed predominantly green fluorescence, indicating that most cells remained viable. In contrast, biofilms treated with CIT8 displayed mostly red fluorescence, consistent with a substantial reduction in biofilm-associated cell viability but retained small regions of green fluorescence corresponding to viable cells. Biofilms treated with CIT8-D exhibited almost exclusively red fluorescence with only minimal residual green signal, suggesting a near-complete loss of viable cells within the established biofilm.

### 3.6 *In vitro* cytotoxicity and therapeutic index of CIT8 and CIT8-D

To extend our previously reported cytotoxicity profile of CIT8 and evaluate the safety of CIT8-D, we assessed cytotoxicity against two mammalian cell lines, human keratinocytes (HaCaT) and human kidney cortex (HKC-8) cells (Figure 5, Supplementary Table S4). Exposure of both cell lines to CIT8 for 24 h at concentrations as high as 128 μg/mL had no detectable effect on cell viability. In comparison, CIT8-D reduced cell viability, with a median lethal concentration (LC₅₀) of 64.6 μg/mL (95% CI, 60.8–69.2 μg/mL) and 74.1 μg/mL (95% CI, 69.2–79.7 μg/mL) for HaCaT and HKC-8 cells, respectively. Accordingly, the concentration-based therapeutic index (LC₅₀/MIC) of CIT8 was >32 on both cell lines, as the LC₅₀ was not reached at the highest concentration tested, whereas that of CIT8-D was 16.2 and 18.5 in HaCaT and HKC-8 cells, respectively.

**Figure 5:**
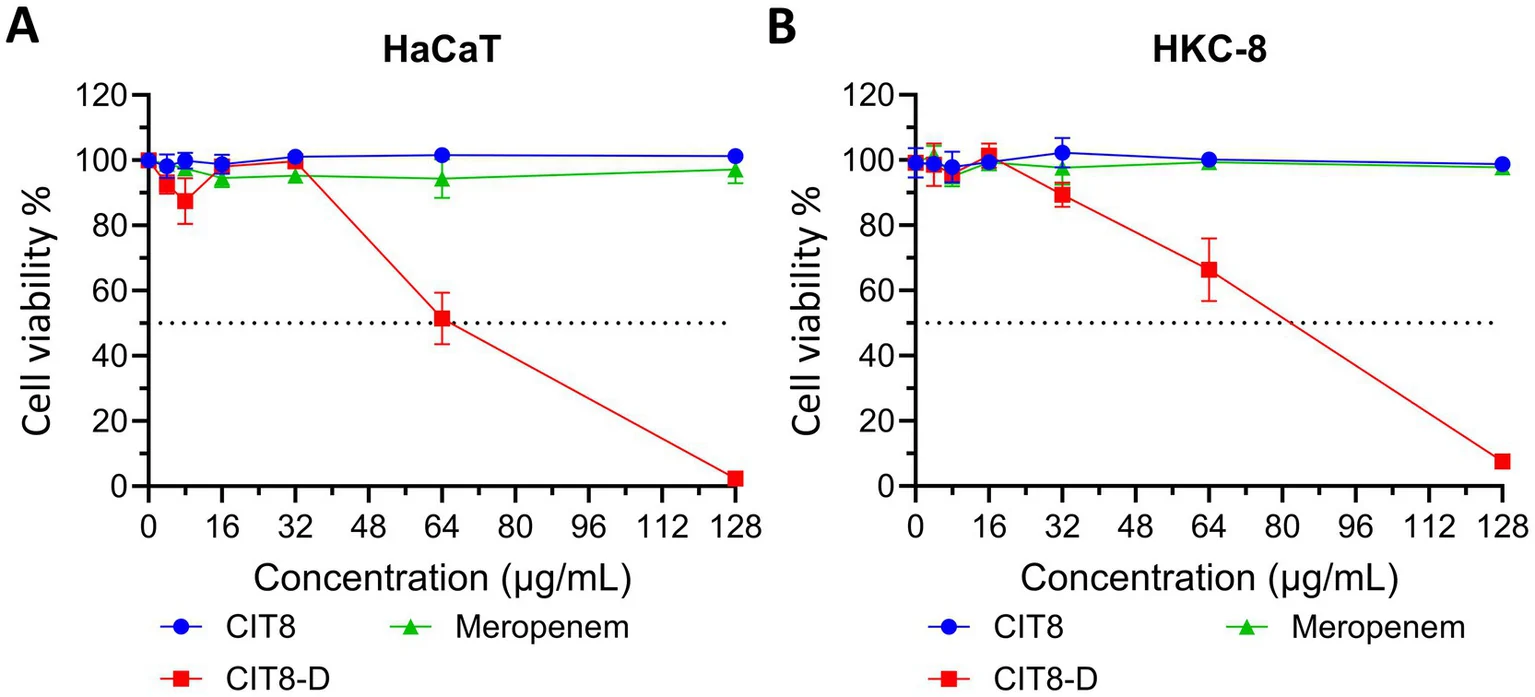
Cytotoxicity of CIT8 and CIT8-D against mammalian cell lines. **(A)** Cell viability of human keratinocytes (HaCaT) following 24 h treatment with the indicated concentrations of CIT8, CIT8-D, or meropenem. **(B)** Cell viability of human kidney cortex (HKC-8) cells following 24 h treatment with the indicated concentrations of CIT8, CIT8-D, or meropenem. Cell viability was determined using the XTT assay. The dotted line indicates 50% cell viability. Data in **(A,B)** are presented as mean ± SD of three independent experiments each performed in three technical replicates (*n* = 3).

### 3.7 CIT8 potentiates the bactericidal activity of meropenem against carbapenem-resistant *A. baumannii*

To investigate whether CIT8 could enhance the antibacterial activity of conventional antibiotics, we performed standard checkerboard assays with clinically relevant antibiotics against *A. baumannii* ATCC19606 and CRAB AR33. However, none of the combinations produced a fractional inhibitory concentration index (FICi) ≤ 0.5, indicating the absence of synergistic interactions under the tested conditions (Figure 6A, Supplementary Figures S6, S7). Considering that CIT8 rapidly disrupts the membrane of *A. baumannii* cells, we next investigated whether it could potentiate the bactericidal activity of meropenem against different CRAB strains. Because standard checkerboard assays assess growth inhibition rather than bactericidal activity, we used a time-kill assay to assess bactericidal interactions, with bactericidal synergy defined as a ≥ 2-log₁₀ CFU/mL reduction relative to the most active compound tested alone (Eliopoulos and Moellering, 1996). Combining 4 μg/mL CIT8 with 40 μg/mL meropenem reduced viable bacterial counts of AR33 by approximately 4 log₁₀ CFU/mL relative to the initial inoculum after 4 h (Figure 6B). This reduction was consistent with the definition of bactericidal synergy, as the combination resulted in an additional 2.7 log₁₀ CFU/mL reduction compared with 4 μg/mL CIT8 alone (****p < 0.0001), whereas meropenem alone produced no measurable reduction. These results were consistent across the other CRAB isolates tested, AR36 and AR63, for which the combination produced additional reductions of 2.0 and 3.2 log₁₀ CFU/mL, respectively, compared with CIT8 alone (Supplementary Figure S8). To further investigate the bactericidal interaction between CIT8 and meropenem, we assessed whether the combination could enhance membrane damage by measuring the leakage of intracellular ATP (Figure 6C). Interestingly, we observed no significant differences in luminescence between the combination-treated groups and the corresponding CIT8-treated groups, suggesting that the combined activity cannot be attributed simply to enhanced membrane damage.

**Figure 6:**
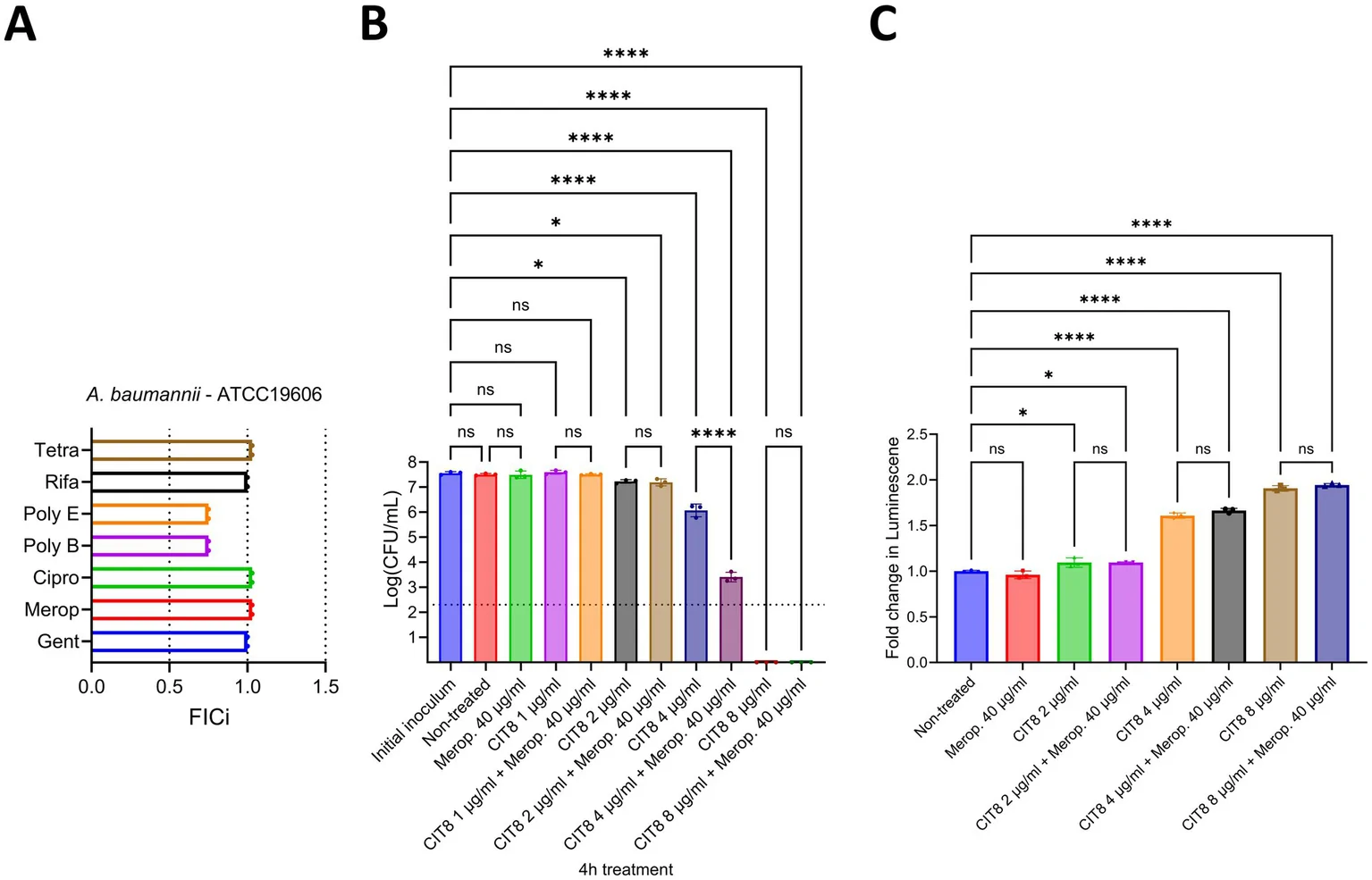
CIT8 potentiates the bactericidal activity of meropenem against carbapenem-resistant *A. baumannii*. **(A)** Fractional inhibitory concentration indices (FICi) for CIT8 in combination with clinically relevant antibiotics against *A. baumannii* ATCC19606. Each data point represents the FICi value from an independent experiment (n = 2 independent experiments). **(B)** Dose-dependent killing of carbapenem-resistant *A. baumannii* AR33 following 4 h treatment with CIT8, meropenem, or their combinations at the indicated concentrations. An untreated group served as the bacterial control. The dashed line indicates the limit of detection (2 × 10^2^ CFU/mL). **(C)** Leakage of intracellular ATP from carbapenem-resistant *A. baumannii* AR33 following treatment with CIT8, meropenem, or their combinations for 1 h. ATP release was quantified using a luciferase-based bioluminescence assay. Data in **(B,C)** are presented as mean ± SD of three independent experiments each performed in three technical replicates (*n* = 3). Statistical significance was determined by one-way ANOVA followed by Šídák’s multiple comparisons test for the indicated comparisons (ns, not significant; *p < 0.05; ****p < 0.0001).

## 4 Discussion

Carbapenem-resistant *A. baumannii* remains one of the most challenging bacterial pathogens to treat largely because of its capacity to acquire resistance and the limited efficacy of current therapeutic options (Bassetti et al., 2021; Kaye et al., 2023; Shields et al., 2023). As such, it is regarded as a top-priority pathogen for the development of new treatment options (World Health Organization, 2024). Although AMPs have shown great promise as an alternative class of therapeutics, identifying and optimizing peptides using conventional experimental approaches is time-consuming and resource intensive. Recent advances in ML have dramatically accelerated these processes; however, experimental validation remains indispensable (Huang et al., 2023; Szymczak et al., 2023; Mishra et al., 2025; Shehadeh et al., 2026; Torres et al., 2026). In the present study, we demonstrate that CIT8, a previously developed ML-optimized derivative of the naturally occurring peptide citropin 1.1, exhibited strong antibacterial and antibiofilm activity against multidrug-resistant *A. baumannii*, including CRAB isolates. Moreover, CIT8 rapidly disrupted the *A. baumannii* membrane without detectable mammalian cytotoxicity and potentiated the bactericidal activity of meropenem against CRAB.

The marked difference in antibacterial activity between CIT8 and its parent peptide, CIT1, demonstrates how relatively small sequence modifications (D4K, V9L, A10L, S11K) can dramatically alter the potency, spectrum of activity, and selectivity of an AMP, consistent with previous studies (Dathe and Wieprecht, 1999; Jenssen et al., 2006; Fjell et al., 2012). Rather than relying on exhaustive experimental screening of numerous peptide variants, the ML-optimization strategy guided the selection of the four amino acid substitutions using a physicochemically distinct cluster (dominated by AMPs with high GRAVY, helicity, and an extended hydrophobic surface) identified from a dataset of more than 14,000 experimentally characterized AMPs with diverse activities against Gram-positive and Gram-negative pathogens (Mishra et al., 2025). Interestingly, although the optimization objective was to develop citropin-based peptides with enhanced anti-MRSA activity, CIT8 exhibited physicochemical characteristics consistently associated with potent activity against both MRSA (Mishra and Wang, 2012) and *A. baumannii* (Neshani et al., 2020; Lima and de Lima, 2023; Rangel et al., 2023; Kumar, 2025).

Compared with CIT1, CIT8 displayed increased net positive charge, hydrophobicity and amphipathicity. Analysis of the intermediate peptide variants (CIT3, CIT4, CIT5 and CIT7) indicated that the enhanced activity against *A. baumannii* was not attributable to a single substitution but rather to the combined effects of these modifications, with CIT8 exhibiting the most favorable combination of antibacterial and hemolytic activity (Mishra et al., 2025). Among these properties, increased positive charge likely contributed directly to the enhanced antibacterial activity by promoting the initial interaction with the bacterial membrane. The outer membrane of *A. baumannii* is rich in negatively charged phosphorylated lipids and lipopolysaccharides (Nikaido and Vaara, 1985; Vinogradov et al., 2002). Consequently, cationic AMPs preferentially bind to the bacterial surface through electrostatic interactions, accumulate at the membrane interface and ultimately disrupt membrane integrity (Powers and Trent, 2017; Lima and de Lima, 2023).

Consistent with a membrane-targeting mechanism, CIT8 induced membrane depolarization, increased Sytox Green uptake, leakage of intracellular ATP and produced pronounced alterations in cell-surface morphology observed by SEM. These membrane-disruptive effects were consistent with the rapid bactericidal activity observed in the time-kill assays, in which viable bacterial counts were reduced below the limit of detection within 15 min. Furthermore, the reduced antibacterial activity of CIT8 in the presence of Ca^2+^, Mg^2+^ and, to a lesser extent, Zn^2+^ highlights the importance of electrostatic interactions during the initial binding of CIT8 to the bacterial surface. Divalent cations are known to stabilize the Gram-negative outer membrane by bridging adjacent lipopolysaccharide molecules, thereby weakening the electrostatic attraction between cationic AMPs and the bacterial surface (Schindler and Osborn, 1979; Nikaido and Vaara, 1985).

All-atom MD simulations provided additional insight into the membrane-binding behavior of CIT8. Although the POPE: POPG membrane model does not capture the complexity of the *A. baumannii* outer membrane, including its LPS-rich outer leaflet, it provides a relevant anionic phospholipid environment for studying peptide-membrane interactions. Both peptides displayed broadly similar membrane-binding behavior and localized predominantly at the membrane interface; however, CIT8 exhibited higher POPG contact occupancy across much of its sequence, suggesting greater interactions with the anionic components of the membrane. Moreover, the conformational stability of the two peptides differed substantially. CIT8 retained a more stable α-helical structure throughout the simulations, while the α-helical structure of CIT1 was less stable and more transient. These findings are in agreement with previous studies (Khandelia and Kaznessis, 2006; Jiang et al., 2008; Gori et al., 2023; Bello-Madruga and Torrent Burgas, 2024; Rangubpit et al., 2025; Catalina-Hernandez et al., 2026) showing that destabilization of α-helical structure is associated with reduced antimicrobial activity, suggesting that the greater α-helical stability of CIT8 may also contribute to its enhanced antibacterial activity.

Beyond their strong activity against planktonic bacteria, both CIT8 and CIT8-D exhibited potent antibiofilm activity against *A. baumannii*. Biofilm formation is a major virulence mechanism of *A. baumannii*, contributing to persistent infections and prolonged survival on abiotic surfaces, such as medical devices and hospital equipment (Bardbari et al., 2017). Within established biofilms, some bacteria often adopt a metabolically inactive or dormant phenotype, substantially increasing their tolerance to environmental stress and conventional antibiotics (Gayoso et al., 2014). Unlike conventional antibiotics, membrane-targeting AMPs can retain activity against these tolerant bacterial populations (Yasir et al., 2018). Accordingly, both CIT8 and CIT8-D markedly reduced the XTT signal when added during biofilm formation and decreased biofilm-associated cell viability in established biofilms, whereas meropenem, while active during biofilm formation, showed little to no effect against established biofilms under the same experimental conditions. In addition, both peptides reduced established AR33 biofilm biomass, although these effects were less pronounced. Notably, CIT8-D displayed greater potency than CIT8 against both developing and established biofilms, but this enhanced activity was accompanied by increased mammalian cytotoxicity. These observations may, at least in part, reflect the greater resistance of D-enantiomeric peptides to proteolytic degradation, which can preserve peptide activity during prolonged exposure (De La Fuente-Núñez et al., 2015; Zhang et al., 2016; Yadav et al., 2025). This prolonged activity may enhance the effects of CIT8-D against biofilm-associated cells while also increasing its cytotoxic effects on mammalian cells, as previously proposed for other D-enantiomeric AMPs (Mardirossian et al., 2017; Varponi et al., 2023). Despite its increased cytotoxicity, CIT8-D maintained considerable selectivity toward bacterial cells, with therapeutic indices of 16.2 and 18.5 in HaCaT and HKC-8 cells, respectively.

CIT8 also potentiated the bactericidal activity of meropenem against CRAB. The combination met the prespecified ≥ 2-log₁₀ CFU/mL threshold for bactericidal synergy in the time-kill assay across all three isolates tested, whereas no synergistic interaction was detected by checkerboard assays. Similar discrepancies between these methods have been previously reported (White et al., 1996; Bonapace et al., 2000; Tan et al., 2011; Heo et al., 2024; Mellett et al., 2025; Bock et al., 2026; Dellis et al., 2026) and likely reflect differences in their experimental endpoints, as checkerboard assays assess growth inhibition based on changes in MIC, whereas time-kill assays capture changes in bactericidal activity over time. Synergistic interactions between membrane-targeting AMPs and β-lactam antibiotics have been widely reported against multidrug-resistant Gram-negative pathogens (Taheri-Araghi, 2024). Perhaps the most notable example is the combination of polymyxins (colistin or polymyxin B) with meropenem, which has been extensively investigated for the treatment of CRAB infections (Zusman et al., 2013, 2017; Qureshi et al., 2015; Lenhard et al., 2017; Nutman et al., 2020). Mechanistically, polymyxins are thought to permeabilize the outer membrane, thereby facilitating the access of meropenem to its periplasmic targets (Lenhard et al., 2017; Ramirez and Schweizer, 2025). Although the precise molecular mechanism underlying this interaction requires further investigation, our findings suggest that CIT8 may facilitate the activity of meropenem against CRAB through a similar membrane-associated mechanism. Nevertheless, further evaluation across multiple concentration combinations is needed to characterize the interaction between CIT8 and meropenem more comprehensively.

While our findings support a membrane-targeting mechanism contributing to the bactericidal activity of CIT8 against CRAB, interactions with additional cellular targets cannot be excluded and require further investigation. In addition, its proteolytic stability, pharmacokinetic properties, and potential for resistance development remain to be determined and represent important considerations for future development. Furthermore, the reduced activity observed in the presence of divalent cations and increasing concentrations of human serum highlights the need to evaluate CIT8 under more physiologically relevant conditions, including higher serum concentrations and complex biological matrices. Beyond these considerations, *in vivo* efficacy and safety studies will be necessary before its translational potential can be adequately assessed.

Overall, our findings demonstrate that relatively small sequence modifications that alter key physicochemical properties can markedly alter the antibacterial spectrum and biological properties of antimicrobial peptides. Although CIT8 was originally developed as an anti-MRSA peptide, it also possesses strong antibacterial activity against multidrug-resistant *A. baumannii*. While further optimization and preclinical evaluation will be required, CIT8 provides a promising scaffold for the continued development of peptide-based therapeutics against multidrug-resistant *A. baumannii*. As ML-driven computational approaches continue to evolve, they may increasingly facilitate the rational identification and prioritization of such structural modifications.

## Data Availability

The raw data supporting the conclusions of this article will be made available by the authors, without undue reservation.
