# Supplementary material for: CIT8, a machine learning-optimized antimicrobial peptide, exhibits potent activity against carbapenem-resistant Acinetobacter baumannii
Source: Front Microbiol. 2026 Oct 19;17:1967486. doi: 10.3389/fmicb.2026.1967486 (PMC13724386; doi:10.3389/fmicb.2026.1967486)
Supplement: Supplementary file 1 [file Supplementary_file_1.docx]

**Supplementary Information (SI)**

**CIT8, a machine learning-optimized antimicrobial peptide, exhibits potent activity against carbapenem-resistant *Acinetobacter baumannii***

Charilaos Dellis^1,2†^, Biswajit Mishra^1,3†^, Liyang Zhang^1^, Anindya Basu^4,5^, Lewis Oscar Felix^1^, Fadi Shehadeh^1,6^, Narchonai Ganesan^1^, Antonios Kolocouris^2^, Eleftherios Mylonakis^1,3*^

**Supplementary Tables**

**Table S1.** Comparative analysis of CIT1, CIT8 and intermediate sequence variants.

| **Peptide** | **Sequence** | **Substitutions relative to CIT1** | **MIC (μg/mL)** | | **HL₅₀ (μg/mL) ^1^** |
| --- | --- | --- | --- | --- | --- |
|  |  |  | MRSA (MW2) ^1^ | *A. baumannii* (ATCC19606) |  |
| CIT1 | GLFDVIKKVASVI | – | >32 | >32 | >128 |
| CIT3 | GLFKVIKKVASVI | D4K | >32 | 16 | >128 |
| CIT4 | GLFKVIKKVAKVI | D4K, S11K | >32 | 16 | >128 |
| CIT5 | GLFDVIKKLLSVI | V9L, A10L | >32 | >32 | 21 |
| CIT7 | GLFKVIKKLLSVI | D4K, V9L, A10L | 4 | 4 | 41 |
| CIT8 | GLFKVIKKLLKVI | D4K, V9L, A10L, S11K | 4 | 4 | 68 |

HL₅₀, peptide concentration causing 50% hemolysis.

^1^ MRSA MIC and HL₅₀ values were previously reported by Mishra et al. (2025).

**Table S2.** Summary of equilibrium molecular dynamics descriptors for CIT1 and CIT8 in a POPE:POPG (3:1) lipid bilayer.

| **System** | **Replicate** | **Distance from bilayer center (Å)** | **Peptide-lipid Hydrogen bonds** | **α-helix content^1^ (%)** | **Orientation angle^2^ (°)** |
| --- | --- | --- | --- | --- | --- |
| POPE:POPG (3:1) + CIT1 | 1 | 22.0 ± 6.0 | 6 ± 2 | 38.2 ± 23.7 | 74.0 ± 13.9 |
|  | 2 | 21.1 ± 2.7 | 5 ± 2 | 17.9 ± 19.2 | 30.2 ± 15.8 |
| POPE:POPG (3:1) + CIT8 | 1 | 19.9 ± 5.7 | 6 ± 3 | 31.7 ± 17.8 | 68.8 ± 14.6 |
|  | 2 | 20.8 ± 6.3 | 6 ± 2 | 64.4 ± 10.5 | 62.5 ± 17.4 |

Values reported as mean ± standard deviation calculated over the 1 μs production trajectory of each independent simulation

^1^ Peptide α-helix content was determined from DSSP secondary-structure assignments using MDTraj

^2^ Defined as the angle between the peptide N-to-C terminal vector and the membrane normal

**Table S3.** Residue-specific interactions with POPE and POPG lipids.

|  | **POPE contact occupancy (%)****^1^** | | **POPG contact occupancy (%)^1^** | |
| --- | --- | --- | --- | --- |
| **aa-position** | **CIT1** | **CIT8** | **CIT1** | **CIT8** |
| 1 | 86.8 ± 8.9 | 71.6 ± 12.5 | 35.0 ± 13.1 | 71.1 ± 5.6 |
| 2 | 80.8 ± 24.5 | 71.7 ± 19.3 | 57.6 ± 4.1 | 73.8 ± 4.2 |
| 3 | 76.4 ± 28.2 | 71.6 ± 19.0 | 62.4 ± 7.5 | 83.6 ± 1.6 |
| 4 | 80.0 ± 2.3 | 71.4 ± 6.4 | 36.0 ± 3.5 | 81.0 ± 7.7 |
| 5 | 77.9 ± 17.4 | 46.0 ± 10.5 | 67.7 ± 10.4 | 67.8 ± 7.3 |
| 6 | 72.7 ± 31.4 | 57.0 ± 14.7 | 67.8 ± 6.0 | 59.0 ± 16.1 |
| 7 | 66.4 ± 33.8 | 45.6 ± 2.6 | 60.8 ± 7.1 | 76.3 ± 4.8 |
| 8 | 75.6 ± 7.0 | 49.4 ± 6.1 | 75.4 ± 6.6 | 74.9 ± 12.8 |
| 9 | 44.8 ± 19.2 | 68.0 ± 14.6 | 55.4 ± 23.1 | 55.6 ± 6.7 |
| 10 | 32.9 ± 19.2 | 58.5 ± 4.1 | 38.8 ± 22.4 | 54.2 ± 2.7 |
| 11 | 48.8 ± 15.4 | 83.1 ± 0.4 | 40.1 ± 28.2 | 64.6 ± 0.3 |
| 12 | 46.7 ± 27.6 | 60.6 ± 22.0 | 29.4 ± 25.9 | 59.7 ± 3.7 |
| 13 | 57.9 ± 26.9 | 50.4 ± 25.7 | 38.2 ± 31.3 | 47.7 ± 13.1 |

^1^POPE and POPG contact occupancies were calculated independently

**Table S4.** Cytotoxicity and therapeutic index of CIT8 and CIT8-D

| **Cell line** | **CIT8** | Therapeutic Index^1^ (LC₅₀/MIC**_CIT8_**) | **CIT8-D** | Therapeutic Index^1^ (LC₅₀/MIC**_CIT8-D_**) |
| --- | --- | --- | --- | --- |
|  | LC_50_ (μg/mL) |  | LC₅₀ (μg/mL) |  |
| HaCaT | >128 | >32 | 64.6 (95% CI, 60.8–69.2) | 16.2 |
| HKC-8 | >128 | >32 | 74.1 (95% CI, 69.2–79.7) | 18.5 |

^1^Therapeutic index was calculated as the ratio of LC50 to the MIC against *A. baumannii* ATCC19606 (LC_50_/MIC)

**Supplementary Figures**

**Figure S1. Concentration-dependent killing of *A. baumannii* by CIT8.** Time-kill kinetics of CIT8 against *A. baumannii* ATCC19606 **(A)** and AR33 **(B)** at 1, 2, 4, and 8 μg/mL (1/4 ×, 1/2 ×, 1 ×, and 2 × MIC, respectively). The dashed line indicates the limit of detection (2 × 10² CFU/mL). Data are presented as mean ± SD from three independent experiments.

 **Figure S2. Residue-specific analysis of CIT1 and CIT8. (A)** Mean distance (Å) of each amino acid residue of CIT1 (light blue) and CIT8 (pink) from the bilayer center, with residues shown from the N- to C-terminus. Smaller values indicate residues located closer to the bilayer center. **(B)** Membrane contact occupancy of each amino acid residue of CIT1 and CIT8, defined as the percentage of simulation frames in which at least one non-hydrogen atom of the residue was within 4.5 Å of a non-hydrogen atom of a POPE or POPG lipid. Data were obtained from two independent 1 μs simulations for each peptide and are presented as mean ± SD (n = 2).

**Figure S3. Potential of mean force for CIT8 interacting with a POPE:POPG (3:1) lipid bilayer. (A)** PMF profile calculated from umbrella sampling simulations using the weighted histogram analysis method (WHAM). Error bars represent the standard deviation estimated from 200 trajectory-based bootstrap replicates with autocorrelation correction. The reaction coordinate corresponds to the distance between the peptide center of mass and the bilayer center along the membrane normal. **(B)** Convergence analysis showing PMF profiles calculated using progressively increasing portions (10–50 ns) of the umbrella sampling trajectories. **(C)** Probability distributions of the umbrella sampling windows along the reaction coordinate, showing overlap between neighboring windows.

**Figure S4. Secondary-structure evolution of CIT1 and CIT8 during 1 μs MD simulations.** DSSP analysis was performed using MDTraj, and the assigned secondary structure of each residue is shown as a function of simulation time. **(A)** CIT1 replicate 1 (left) and replicate 2 (right). **(B)** CIT8 replicate 1 (left) and replicate 2 (right). Colors indicate the DSSP secondary-structure assignment for each residue, including α-helix (red), 3₁₀-helix (pink), π-helix (orange), β-strand (purple), β-bridge (lavender), turn (light orange), bend (dark grey), and loop/irregular (light grey) conformations.

**Figure S5**. **Effects of CIT8 and CIT8-D on established *A. baumannii* AR33 biofilm biomass.** Established *A. baumannii* AR33 biofilms were treated with CIT8, CIT8-D, meropenem, or polymyxin B at the indicated concentrations for 24 h. Remaining biofilm biomass was quantified by crystal violet staining and expressed as a percentage of the untreated control. Data are presented as mean ± SD from three independent experiments.

**Figure S6. Combination of CIT8 with selected antibiotics against *A. baumannii* ATCC19606. (A–G)** Checkerboard microdilution assays of CIT8 combined with gentamicin **(A)**, meropenem **(B)**, ciprofloxacin **(C)**, polymyxin B **(D)**, polymyxin E **(E)**, rifampin **(F)**, and tetracycline **(G)** against *A. baumannii* ATCC19606. Heat maps indicate bacterial growth, with dark red corresponding to higher densities. Synergy was defined as FICi ≤ 0.5. Data are shown from a representative experiment. Experiments were independently repeated twice.

**Figure S7. Combination of CIT8 with selected antibiotics against CRAB AR33. (A–G)** Checkerboard microdilution assays of CIT8 combined with gentamicin **(A)**, meropenem **(B)**, ciprofloxacin **(C)**, polymyxin B **(D)**, polymyxin E **(E)**, rifampin **(F)**, and tetracycline **(G)** against carbapenem-resistant *A. baumannii* AR33. Heat maps indicate bacterial growth, with dark red corresponding to higher densities. Synergy was defined as FICi ≤ 0.5. Data are shown from a representative experiment. Experiments were independently repeated twice.

**Figure S8. Bactericidal interaction between CIT8 and meropenem against CRAB isolates AR36 and AR63**. Dose-dependent killing of carbapenem-resistant *A. baumannii* AR36 and AR63 following 4 h treatment with CIT8, meropenem, or their combinations at the indicated concentrations. An untreated group served as the bacterial control. The dashed line indicates the limit of detection (2 × 10² CFU/mL). Data are presented as mean ± SD from three independent experiments. Statistical significance was determined by one-way ANOVA followed by Šídák’s multiple comparisons test for the indicated comparisons (ns, not significant; ***P* < 0.01; ****P* < 0.001; *****P* < 0.0001).
